# “You get tired of remaining in a state of fear“- professionals’ experiences of self-care facing suicidality in psychiatric wards

**DOI:** 10.1080/17482631.2023.2292184

**Published:** 2023-12-19

**Authors:** May Elise Vatne, Vibeke Lohne, Dagfinn Nåden

**Affiliations:** Oslo Metropolitan University, Faculty of Health, Department of Nursing and Health Promotion, Oslo, Norway

**Keywords:** Suicide behaviours, suffering, psychiatric wards, professionals’ experiences, self-care, hermeneutics

## Abstract

**Aim:**

The aim of this study is to explore mental healthcare professionals (MHCPs) experiences related to own emotions when encountering patients at risk of suicide in psychiatric wards and their family members.

**Methodology and methods, participants and research context:**

This study has a qualitative explorative design. Data consist of texts from twelve in-depth interviews with MHCPs belonging to six units in two psychiatric wards. Data were interpreted using a hermeneutical approach based on Gadamer’s philosophical hermeneutics.

**Findings:**

Through an interpretation process, three themes emerged: Enduring own emotions, Balancing emotional engagement and the need to rest, and Being together in the community of colleagues.

**Conclusion:**

This study shows the importance of being aware of own anxiety facing suicidality. MHCPs have to work emotionally and cognitively so that care is not guided by anxiety but by collaboration with the patient and his family members. The study highlights the need for a culture in the mental health service in which the MHCP can reflect on own emotional reactions and thoughts in a collegial environment characterized by openness, generosity and collaboration.

## Introduction

Every year, 703 000 people in the world die by suicide. The suicide affects families, communities and entire countries and has long-lasting effects on the people left behind. Many more people attempt suicide (WHO, World Health Organization, 2023, August 28). Mental healthcare professionals (MHCPs) who work as milieu therapists in psychiatric wards provide 24-hour care and encounter suicidal patients’ unbearable suffering. Communication and relationships are key components emphasized in MHCP`s specialization. A high proportion of patients admitted to the acute psychiatric wards have contemplated or attempted suicide, and assessment of suicide risk is a key task in such wards (Mellesdal et al., 2010). Caring for suicidal persons is predicated on engagement that could inspire hope (Cutcliffe & Barker, 2002; Cutcliffe & Stevenson, 2008). The family members are usually a central resource for the patients at suicidal risk and need to be involved. Engagement that could inspire hope is important for the patients’ family members too, who need care themselves in order to be a resource for the patient. Caring for suicidal patients is emotionally demanding (Cutcliffe & Stevenson, 2008) and is described by Hagen et al. (2017a) as one of the most challenging caring situations for health care professionals. MHCPs also encounter patients’ family members who feel tired after living with worry and in a state of constant anxiety, and after struggling with helplessness over not being able to help a suicidal person who is close to them (McLaughin et al., 2014).

This study deals with MHCPs’ experiences in taking care of themselves in their encounters with patients in psychiatric wards at risk of suicide and their family members.

## Background

The theoretical framework in this study is Katie Eriksson’s caring theories. According to Eriksson, suffering is an aspect of the human being and affects the courage to live (Eriksson, 2006, 2018, 2022). Suicide attempts and suicide is understood as a result of intense psychological pain which is experienced as intolerable suffering (Shneidman, 1998; Williams, 2014). The experience of suffering can also give rise to growth (Eriksson, 2018). According to Cutcliffe et al. (2015), suffering needs to be endured, minimized, relieved, explored for meaning and maybe even learned from. This process requires connection to those suffering (Cutcliffe & Barker, 2002), and, according to Eriksson (2006, 2018), to be aware of the other’s pain or suffering. In line with Eriksson, health means wholeness and holiness and is understood as a state of perceived health and well-being while not necessarily in the absence of suffering. Care and health are part of human nature. The aim of care is to promote and protect health and alleviate suffering. Vitality is the innermost dimension of health; it is a source of energy in life, of joy and desire (Bergbom et al., 2022; Eriksson, 1984, 2018).

Emotions such as fear and sadness are understood as reactions to experiences. In order to meet challenges in the encounter with patients at risk of suicide and their family members and to provide good care, it is crucial to be aware of one’s own emotions. Encountering is an act of care where two individuals listen to each other so that loneliness is transformed into communion (Koskinen & Lindström, 2015; Lindström, 1994), which in turn can facilitate movement towards health. Cutcliffe and Stevenson (2008) point out that caring for a suicidal individual must be an interpersonal endeavour which is created by talking and listening in a care relationship.

Previous research that sheds light on professionals’ experiences related to suicidality mainly focuses on the impact patient suicide has on them personally and professionally. A substantial number of mental health professionals have experienced the suicide of a client. Dransart et al. (2017) indicate that 55% of nurses and 33% of social workers have had such an experience. Hendin et al. (2000) point out that losing a patient to suicide is often described as the most disturbing event in a health professional’s career.

Most patients at risk of suicide do not commit suicide. Studies show that patients’ suicidal intentions cause therapists more stress than any other behaviour or communication (Hendin, 2000; Dransart et al., 2017; Gulfi et al., 2010). Facing suicidality evokes a wide range of emotions and reactions in healthcare professionals such as anxiety, helplessness, anger, guilt, frustration, concern and fear of accusations (Alhamidi & Alyousef, 2022; Scupham & Goss, 2020; Türkles et al., 2018; Hagen et al., 2017a; Vråle, 2014; Talseth & Gilje, 2011). Anxiety was found to be related to feelings of professional and personal vulnerability and caregivers’ need to protect patients from suicide as well as protect themselves. Professionals fear that a successful suicide can result in their own “professional death” (Morrissey & Higgins, 2019).

Pressure of work and lack of time is found to be factors with negative impact on the professional’s ability to care for themselves when working with patients at risk of suicide (Scupham & Goss, 2020). Further psychotherapeutic training, supervision as well as increased reflexivity can protect mental healthcare professionals and also help them to regulating themselves emotionally and then working more creatively and collaboratively with the suicidal patient (Hagen et al., 2017a; Cutcliffe & Stevenson, 2008; Talseth & Gilje, 2011; Morrissey & Higgins, 2019; Alhamidi & Alyousef, 2022). Sharing burdens of being affected by a patient at risk of suicide with colleagues is a way to balance emotional involvement and professional distance (Hagen et al., 2017a).

The importance of support for professionals working with patients at risk of suicide is often emphasized in previous research. To our knowledge, research with a focus on the professionals’ own experiences of support is scarce in the field of mental health care and even less in cases of encounters that include these patients’ family members.

### Aim

The aim of the study is to explore MHCPs’ experiences related to own emotions when encountering patients at risk of suicide and their family members.

The research question is: What do MHCPs perceive as crucial in taking care of themselves in encounters with hospitalized patients at risk of suicide and their family members?

## Methodology and methods

This qualitative research has a hermeneutic approach with an explorative design. Gadamer’s philosophical hermeneutics (Gadamer, 2004) has been applied as a guide in the encounter with both the participants and the texts; hermeneutics also forms the basis for the interpretation of opinions related to reaching a deeper understanding of the matter. A hermeneutic approach requires a conscious awareness of one’s own prejudices and pre-understanding. Hermeneutics is characterized by sensitivity and receptiveness to what reveals itself in the process of discovery, which in turn can engender new questions so that existing understanding is challenged and expanded (Gadamer, 2004; Nåden, 2010). Understanding is about understanding the matter differently, by reading texts from the part to the whole and back to the part (Gadamer, 2004).

### Recruitment, context and participants

This study is a part of a larger project with focus on MHCPs’ experiences when a patient at risk of suicide is hospitalized in a psychiatric ward in cases where they also have a relationship with the patient family members.

A request to interview health personnel was sent to the senior clinical leader in two hospitals having acute psychiatric functions. The leader made sure that everyone who met the criteria for participation received information about the project and thereby their interest could be registered. The criteria were: Nurses and others with a three-year health and social work background and with specialized studies in mental health care (1), both men and women (2), a minimum of five years’ experience in mental healthcare (3) and in having contact with patients at risk of suicide and their family members (4). One of the researchers received the names of interested parties either from employees themselves or from one of the staff members who had a role as coordinator.

This study included twelve professionals—three men and nine women. These were recruited from five acute units and one subacute psychiatric unit. They all had lengthy experience in the mental health field (see Table I) and from caring with hospitalized patients at risk of suicide. Most of them had the longest experience from the hospital where they were employed when the interviews took place. In addition to specialized studies in mental health care, several of the participants had further education in other fields such as family therapy and group therapy. Nine of the participants worked as milieu therapists while three others possessed different special functions.

**Table I.** Characteristics of the participants.

Years of work experience in mental health service: 5–22 (§ 15)
Current place of work: Six units in psychiatric wards in two hospitals, of which five acute units and one subacute unit
Profession:
Nurse (Eight) Social worker (Three) Social educator (One)
Gender:
Men (Three); Women (Nine)

§=The average sum of experiences in mental healthcare.

### Data collection

The data material consists of texts from individual research interviews conducted at the participants’ workplace, audio-file recorded and transcribed by a company certified to do this. The interviews lasted between 60 to 90 minutes each. The interviews were prepared and carried out in accordance with Kvale’s conception of the interview as a research conversation. The interviews were based on an interview guide to make the conversation planned and flexible in order to facilitate an open and dynamic dialogue and thereby a rich body of material (Kvale & Brinkmann, 2009).

The opening question invited the participants to talk about a situation involving a patient they have had in their care expressing suicidal intentions or having attempted suicide, and with one or several family members being involved in the treatment. Although the object of the main study was experiences in the encounter with family members of suicidal patients, the narratives alternated between relations with the patient and with their family members. The participants were asked about what they perceived were special challenges in such situations. A main question in the interview situations was how they dealt with their own emotional reactions related to encounters after a suicide attempt or with patients at risk of suicide and with their family members. This interview question was formulated after a dialogue with three leaders in one of the two psychiatric wards where the participants were recruited from, before the interviews were conducted, and illustrates a fusion of the authors’ and leaders’ horizons.

### Data interpretation

The researchers individually read all interviews to familiarize themselves with the texts and focus on the participants’ thoughts and reflections. The interviews were then read and reread by the researchers as a group in order to share ideas about potential recurrent themes that could illuminate the research question. In this phase, text passages deemed appropriate to document various themes were marked. The content, meaning the responses to the questions in the interview guide, were presented in an early phase in the interpretation process in the resource group affiliated with the project. The task of this group is explained in the section below under the heading Strengths and limitations.

Texts from the twelve interviews, amounting to 360 A4 pages, constituted a rich data material which we assessed as fully valid to illuminate the research question, and led to data saturation. Data were further interpreted in a search for themes through dialogue with the text. During the interpretation process and while the work of presenting findings was going on, the themes became clearer. In this phase, we went back to the interviews to check whether the quotations documented the identified themes. Reflexivity involves recognizing that one’s pre-understanding affects the research process and thereby the formulation of the themes. Table II illustrates the process of searching for potential themes leading up to the three themes which constitute the findings of this study. A hermeneutical approach to the interview texts, according Gadamer (2004), implies a receptivity to a different understanding, until the interpretation is adopted as consistent and the selected quotations seem to give meaning to and substantiate the themes.

**Table II.** Phases in the interpretation process.

Examples of quotations	Searching for potential themes	Themes
– That’s the worst thing really, when they are on leave and don’t return at the agreed time … everyone is a little stressed by it//In a way, you have to allow yourself to be a little stressed//I get anxious//It is not good to be anxious, but it is good at any rate to acknowledge it.	– Recognize your own anxiety and fear of suicide – Watch to give responsibility back to the patient himself	Enduring own emotions
– it’s important to have that contact, relationship, and you’ll need to spend time building it. But, on the other hand, you shouldn’t spend too much time, because then you will wear yourself out and be incredibly exhausted from always being in a state of uncertainty.	– Spend time on building the relationship in cases involving a risk of suicide – Be emotionally accessible, but acknowledge the need for a break from responsibility	Balancing emotional commitment and the need to rest
– It is all right to say, “I’m very tired of this now”, and then hear a colleague say “Yes, I understand, because I’ve been there during the past weekend” - that way you know that you both share the same feeling. It lays the foundation for a shared experience, and that feels good.	– Experience openness, generosity and collaboration in the working environment – Find support in the fact that colleagues recognize their own reactions	Being together in the community of colleagues

### Ethical considerations

The participants were especially cautious about not identifying patients, family and staff members by name and personal details. A total observance of the duty of confidentiality will always be a challenge in research involving questions about personal experiences. Information that might help identify persons was therefore either omitted or anonymized, even at the risk of losing some of the meaningful content. In research interviews, some moral responsibility is also a given necessity. We invited the participants to share personal experiences and reflections in openness and trust, which requires the researcher to be sensitive to the participant’s integrity and dignity.

The study was approved by the Ombudsman for Privacy of the Norwegian Social Science Data Services, (no. 54982/2017). The participants gave written consent, based on written and oral information about the project, including anonymization and the right to withdraw from the study.

## Findings

Three themes emerged from the text: Enduring own emotions, Balancing emotional engagement and the need to rest, and Being together in the colleague of community. These themes illuminate what the mental healthcare professionals who participated in this study experience as crucial in taking care of themselves when encountering hospitalized patients at risk of suicide and their family members. The need of self-care is understood as a result of involvement and engagement in both the patient and his/hers family members. The findings are presented by selected quotations to illustrate and document the themes.

### Enduring own emotions

The participants described relating to a patient at risk of suicide as tiring, but also as challenging and exciting. Experience helped them trust risk assessments that had been made. The fear of suicide was a recurrent theme when participants talked about their own reactions and was particularly linked with experiences involving patients with long-term suicidal behaviour and several suicide attempts and who they feared might eventually succeed in committing suicide. One participant described the constant worry and apprehension as follows: “It’s a feeling of always being tensed up at work and really not being secure.”

Working within a system that demanded close follow-up of a patient at risk of suicide increased the participants’ sense of security. In situations where responsibility was gradually given back to the patient himself, such as being given a leave of absence, one participant related how the ability to tolerate his or her own emotions was put into play:

… That’s the worst thing really, when they are on leave and don’t return at the agreed time … everyone is a little stressed by it, but at the same time we can talk to each other and ask one another “What do we do now?” In a way, you have to allow yourself to be a little stressed. And then you have to develop some strategies. I get anxious. And that is really a little … It is not good to be anxious, but it is good at any rate to acknowledge it. (Participant no. 7)

The participants said that they spent a lot of time with the patients’ family members to address their reactions as anxiety, worry and frustration. They drew parallels between their own experience with fear and the experiences of the patients’ family members. “I think apprehension and fear are a common denominator for relatives,” one participant said. The participants pointed out that the family members’ experiences were at a completely different level compared to their own, because professionals didn’t have the same relationship as the relatives either before or after discharge, and they also had a different network around them, due to being health care professionals. “But being able to draw parallels makes us better able to accommodate the needs of relatives”, as one participant put it.

To endure fear without taking on the responsibility for ensuring the patient’s survival was described as a challenge. Several participants reported that they reflected on their own lives in conversation with parents of young people at risk of suicide about their fears. In situations involving over-involvement on the part of parents, one of the participants reflected on what they might have done: “I don’t think I would have left my child to herself either.”

It is important to be “emotionally open” when you are in a relationship involving patients at risk of suicide and their family members, one of the participants said. They emphasized the importance of recognizing their own feelings, but without being overwhelmed by them: “You must act and do what is best in the situation at hand,” another said, and added:

The degree of powerlessness you feel in the situation also varies a little. There are so many suicidal inclinations here, so I really feel that we can handle a lot of it … many times family members are very frustrated, and sometimes you feel the need to justify the system to them a little. Sometimes it may be appropriate, and other times it is inappropriate, of course. (Participant no. 7)

The importance of recognizing and daring to make clear what the staff can do and what they cannot take responsibility for, both for patients and their family members, was described as follows by a participant:

One of the things we can’t take responsibility for is guaranteeing that a person will stay alive. We can take responsibility for providing optimal treatment … but we cannot take on that one responsibility. Because if one takes the responsibility for things that one cannot possibly be held accountable for, you will totally exhaust yourself. Just like patients’ family members do. (Participant no. 1)

### Balancing emotional commitment and the need to rest

Participants underscored the importance of relationship-building and spending time as perhaps the most important factors in preventing suicide. They invested time and were fatigued, but not worn out. “To gain the patient’s confidence, you have to work hard at it, talk a lot and spend time together,” this participant said:

So I think there are dilemmas, because it’s important to have that contact, relationship, and you need to spend time building it. But, on the other hand, you shouldn’t spend too much time, because then you will wear yourself out and be incredibly exhausted from always being in a state of uncertainty about what state the patient is in when you come into their room. (Participant no. 11)

To be able to provide good care to patients at risk of suicide and their family members, we need to make sure we stay on the job for many years and that our goal is “to hold out”, the participants expressed. Because, as one of them said, “the patients have needs, and family members have needs. But if we are unable even to take care of ourselves, then we won’t be good helpers”.

The participants emphasized the importance of balancing emotional closeness and distancing so that they do not “fill themselves up” with their own feelings and lose their scope of action. The fact that a colleague relieves them by taking over responsibility when a patient is on constant watch because of an acute risk of suicide helped them to get needed “breaks”:

I think variation is very important, and that you have enough people on the team so that you don’t have to remain a hundred percent with the same patient over a period of several weeks. If you remain very close to the same patient over time, you will completely exhaust yourself. In addition, you get tired of remaining in a state of fear that you almost every day might lose patients. (Participant no. 11)

Participants talked about situations involving long-term relationships with patients at risk of suicide that took so much energy that their private lives were affected. One participant talked about having reduced capacity to socialize during the period when thoughts about a suicidal patient preoccupied the participant:

You notice that you need more “space”. I don’t have the same energy, perhaps. I have much more of a need for peace and quietness because there is so much happening (with a suicidal patient), so much to think about… that I don’t always want to talk on the phone or meet people… (Participant no. 11)

This participant talked about a relationship with a suicidal patient who became very intense in one phase and where the participant’s fatigue resulted in frustration, exemplified as follows: “But we had an agreement on this, … didn’t we say that you were not going to do that now? … but we really couldn’t count on it, could we?” Situations where the patient is unable to keep an agreement are perceived as demanding when one is too tired, the participant said: “I find that I get both annoyed and frustrated.”

The participants claimed that lengthy work experience made it easier to walk away from responsibility at the end of the workday. “The job generates less anxiety in me than before, but without offering any guarantee that suicide won’t occur,” as one participant said. However, even with lengthy experience with patients at risk of suicide, situations arose where participants had trouble in leaving worries behind when the workday is over. One participant reported that increased workload at home after having her own children made it easier to leave the job at the workplace.

The normal workday was described as meaningful and varied, but that without “replenishing themselves”, their own energy declined. Some of the participants emphasized the importance of good private relations that allowed them to share anonymous experiences. Others had experienced renewed energy through physical activity, by enjoying nature and music, and in general doing things that they found mentally and bodily replenishing.

### Being together in the community of colleagues

The participants emphasized fellowship in a working environment characterized by openness, mutual generosity and collaboration as supportive for enduring emotional pressure from relating to patients at risk of suicide. And: “One encounters some frustration from patients’ family members, as well … you become a kind of “rubbish bin”, but it is sort of understandable,” one of them said, adding:

It is terribly tiring at times; it is. But I like it a lot, even so. It is a very good working environment, fortunately. It is the very reason why one feels like continuing to work, I think. (Participant no. 5)

“We talk a lot together” was a recurrent comment. The participants said that in such conversations, differing views on the patient’s suicidal status might be expressed, or the fact that several staff members had observed the same phenomenon. As one of them put it:

I think it’s a milieu-therapeutic reflex I have, that one should talk about things, and that it’s both fun and stimulating… It is the community that perceives and provides the foundation to build on going forward. (Participant no. 6)

The participants felt it was easy to talk to a colleague when the need arose and also easy to go to the patient’s main caregiver “in a period of stormy gales. No one will ever laugh at you because you’re worried. And people are worried in different ways,” as one of them said. An example given was situations when patients on leave do not answer the phone and a colleague becomes very anxious, to the point where they are unable to help her “calm down”. “We have a culture of care for one another,” one of the participants said. One needs to be seen in order to remain in demanding relationships and situations involving potential suicide. As one participant said:

In a way, there is recognition and comfort in it. It is all right to say, “I’m very tired of this now”, and then hear a colleague say “Yes, I understand, because I’ve been there during the past weekend” – that way you know that both of you share the same feeling. It lays the foundation for a shared experience, and that feels good. (Participant no. 11)

The participants were aware of the burden family members struggled with by living close to a suicidal person. “They don’t have the network we as professionals have”, as one said. The participants reported that they had staff meetings in which they could talk about demanding cases and situations. They had experience with supervisory personnel taking responsibility for organized conversations after self-harm incidents, suicide attempts and successful suicides. A participant reported that one theme in such meetings is the “pitfall” that the team can fall into on the day a patient takes his or her life, and ways they can collaborate “without trying to find a scapegoat”. The participants experienced that they have good rapport. “Also, I have always thought that my colleagues who take over the shift when I go home do a good job”, one participant said. Several participants mentioned regular guidance in groups as an offer that is important. What was primarily described as significant were the more spontaneous conversations with one or more colleagues that they knew well and whom they trusted were able to reach out to them when the need arose to share thoughts about a suicidal patient.

## Theoretical interpretation and discussion

Being in a relationship to patients with suicidal disorder is understood as being in a state of constant preparedness. Patients’ communication of suicidal intentions triggers more intense reactions and more stress among healthcare professionals than other types of behaviour or communication, and the anxiety of potential suicide lies latent as the greatest fear of health personnel (Hendin, 2000; Gulfi et al., 2010). The theme *Enduring own emotions*, shows the importance of recognizing one’s own emotions and stress in terms of suicide-related patients and their family members. Koskinen and Lindström (2015) define listening as a caring act. In parallel to being attentive to the meaning of expressions of suffering from the patient and his or her family members, MHCPs must listen to their own emotions and what these are all about, to maintain their own health and professional integrity. Experience from this study confirms that patients’ contemplations, plans and attempts to take their own lives affect MCHPs both personally and professionally. The fear that a patient admitted to a psychiatric unit will take his own life can be related to the commitment and need to protect the life of the other person, as well as the need to protect oneself (Morrisey & Higgins, 2019). Caring for a patient at risk of suicide requires the ability to endure and embrace one’s own fears. In the encounter with family members of patients at risk of suicide, it is considered a central task of MHCPs to embrace their anxiety and exhaustion (Vatne et al., 2021). By knowing and having felt the fear of suicide oneself, it seems easier to empathize with and understand the situation and needs of the patient’s family members. Being affected by their suffering may stimulate one’s ethical responsibility to alleviate it (Vatne et al., 2021). To let ethics be the guiding star in the encounter with a suicidal mindset is to act in conformity with Eriksson’s mantra: “I was there, I saw, I witnessed, and I became responsible” (Eriksson, 2018).

Suicide is an event that triggers internal and external scrutiny. The fear of making mistakes that can be criticized by family members, colleagues and health authorities will therefore create an increased vigilance. Some 55% of nurses and other environmental personnel will experience losing a patient to suicide during their career (Dransart et al., 2017). Morrissey and Higgins (2019) found that some nurses shielded themselves from their own anxiety in the encounter with suicidal patients by applying known risk assessment methods and controlling the patient’s behaviour in the form of constant observation. Others, using theory and guidance, were able to keep their own fears in check and give the patient increased responsibility even though this felt risky. Similarly, Hagen et al. (2017b) identified two approaches to suicidal behaviours: “connection and care” and “duty and control”, the latter of which corresponds with what the participants in this study described. Experience seems to be helpful in enabling one to tolerate and explore the suffering of patients, relatives and oneself. In line with Eriksson (2018) and Cutcliffe et al. (2015), exploring the meaning of one’s own anxiety, helplessness and irritation will present learning opportunities. Such exploration requires contact with oneself and one’s inner core (Bergbom et al., 2022). By working with your own emotions in the face of suicidal suffering, relationships that demand a great deal of energy can also add energy and provide professional and personal learning and growth. According to Eriksson (1984, 2018), liberated vitality can provide power to human energy.

Caring for oneself in relationship with patients at risk of suicide and their family members requires *balancing emotional engagement and the need to rest;* identified as a theme in this study. To understand the suffering of suicidal behaviour, the professional must talk to the patient and ask what causes the suffering; about feelings, concerns and pain (Shneidman, 1998; Williams, 2014). When care focuses on the relationship, direct questions about suicide can be experienced as stimulation to share thoughts that in themselves can contribute towards relieving suffering. At the same time, those posing the questions must embrace thoughts and feelings that allow them to suffer with the other person. The patient as well as his/her family members must experience being seen and confirmed in their suffering (Bergbom et al., 2022; Eriksson, 2006, 2018). The investment in time to create a relationship with patients at risk of suicide and their family members is perceived as demanding but at the same time meaningful. The approach seems to be in line with what Cutcliffe and Barker (2002) argue in favour of: Engagement and inspiring hope as the main principle in caring for patients at risk of suicide. At the same time, MHCPs must maintain sufficient distance so as not be overwhelmed by the patient’s hopelessness but be able to communicate hope to the patient and family members without under-communicating the seriousness of the risk of suicide (Vatne et al., 2021). Professionals in the psychiatric wards constantly deal with this responsibility, which can be experienced as burdensome (Vatne, 2006). To remain in the relationship without distancing themselves emotionally and at the same time taking care of themselves, professional caregivers need a rest from responsibility. Specifically, this involves pauses or breaks in their contact with the patient, with a colleague intervening and relieving the caregiver of responsibility. Professionals are affected by concern and anxiety about the patient’s life and by being in a state of psychological preparedness, as family members are. A study shows that MCHPs advise family members to take breaks and do something for themselves when their near kin at risk of suicide are admitted, when they see that the relationship and situation detract from their own needs and health (Vatne et al., 2021). This study shows that professionals seem to give advice based on experience pertaining to what helps them even when they are in an intense relationship with a patient at risk of suicide, and this serves to make the advice credible. At the same time, being attentive and present are necessary so that they examine the family members’ situation and needs separately from their own. This study documents the fact that relationships with patients at risk of suicide can be so demanding and intense that they also affect MCHPs’ private lives. One way to regulate one’s own feelings is to share thoughts and feelings with colleagues, as Hagen et al. (2017a) also found, which enables the caregiver to be close to the relationship and at the same time maintain enough distance to act ethically, professionally and responsibly. However, work pressures and lack of time may increase stress and have a negative impact on self-care and professional functioning in the longer term, as pointed out by Scupham and Goss (2020). Caring with a focus on one’s relationship entails a real encounter and will contribute to compromising one’s own comfort (Gustin, 2012; Lindström, 1994). To suffer with another requires emotional presence, not only for the other, but also with and for oneself. Ethically based care includes care of the other and care of oneself (Bergbom et al., 2022; Eriksson, 2018). According to Gustin (2012), presence and openness are aspects that help professionals take care of themselves in demanding situations.

A central theme in this study is *Being together in the community of colleagues* and deals with experiences of belonging to a mutual, professional environment. Being able to share with colleagues, rather than having to bear the burden of emotional stress alone after encounter with patients at risk of suicide and their family members, is emphasized as an important factor in taking care of oneself and further developing one’s competence. Experience from this study highlights in particular the importance of the community of colleagues to maintain commitment to and satisfaction with one’s job. Sharing thoughts with colleagues who recognize their emotional stress, and confirming them, appear to be comforting and are in line with what Alhamidi and Alyousef (2022) found in their study. Increased knowledge, training and guidance (Cutcliffe & Barker, 2002; Talseth & Gilje, 2011; Hagen et al., 2017a) and support from and collaboration with colleagues seem to be essential for remaining in relationships with suicidal patients and for working with their own feelings and reactions, and to prevent fatigue from leading to health problems.

Findings in this study highlight the importance of the workplace culture for the individual to maintain engagement, energy and well-being in working with patients at risk of suicide. A working environment characterized by openness, attentiveness, respect, generosity and collaboration stands out as a prerequisite for staying on the job over time. Morrissey & Higgins (2019) point out that without making provisions for having a culture in which their own fear of suicide risk is addressed, many professionals will seek to shield themselves from anxiety rather than work to cope with it. In our view, such a culture must be built up and worked on continuously, with staff leaders playing an important role. To grow, caregivers must find their own approach through experience in encountering patients with suicidal tendencies. As Cutcliffe and Stevenson (2008) point out, caregiving for suicidal patients is a human endeavour accomplished through talking and listening. Similarly, caring for oneself comes about by being attentive to one’s own emotions and by reflecting on experiences in a community of colleagues. Listening and reflection are perceived as important sources for health and professional growth in the encounter with patients’ and family members’ suffering. The study shows that caregivers, despite being tired, experience the encounter with patients at risk of suicide and with their family members as meaningful and rewarding. Such encounters yield vitality in line with Eriksson’s view on health: When we experience doing something valuable, our experience of having value as a human being is also affected (Bergbom et al., 2022; Eriksson, 1984, 2018).

## Strengths and limitations

The participants represented both men and woman and belonged to six units in two hospitals. The average sum of experience in mental health was 15 years. Perhaps participants with less experience and from several hospitals could have added more nuances to the themes.

The researchers’ own preunderstanding may represent an obstacle. But according to Gadamer (2004), preunderstanding also represents a positive premise for a different understanding. Although we argue that the first author’s preunderstanding through working with suicidal patients in a context of mental health prevention has a positive impact on the conduct of the study, it is crucial to be critically aware of one’s preunderstanding throughout the research process.

Data in this study were created in an atmosphere characterized by openness and trust. Based on content and depth, data were considered rich enough to answer the research question. Findings in this study are considered valid and reliable, based on the chosen methodology and theoretical perspectives. In line with Kvale and Brinkmann (2009), to validate is to reflect and control through all stages in the research process.

The project was led by the first author, who also conducted the interviews. The co-authors participated in the project from planning to publication of the results. The project has had an external resource group consisting of participants representing patient and user experiences, family member’s experiences, clinical experiences and research. The group consented to follow the project and have offered input via regular meetings with the researchers.

## Conclusion

Suicide is an experience that healthcare professionals in general fear. On a psychiatric unit, MCHPs relate on a daily basis to suffering of patients at risk of suicide and the suffering of their family members. This study confirms that encounters with suicidal behaviour emotionally affect mental health care professionals (MHCPs) and trigger emotional reactions. One reaction that is provoked in particular is the fear of suicide that MCHPs must cope with both emotionally and cognitively in order to empathize and provide good care. The importance of recognizing and enduring one’s own emotions, in addition to the willingness to work to cope with them, is perceived as a prerequisite in providing good care, as well as developing oneself as a professional.

Care of patients at risk of suicide and their family members must be guided by listening, dialogue and collaboration. In this demanding work, MHCPs need to be seen and confirmed to be able to take care of themselves as well. Being in an intense relationship over time necessitates creating breaks in the contact with patients to relieve emotional stress. To take care of oneself requires a working environment conducive to talking about feelings and thoughts about suicide. To both build and maintain a working climate characterized by openness, generosity and collaboration requires active leadership. The experience of community and collaboration with colleagues can be of crucial importance to the individual’s work in safeguarding their own health and developing themselves professionally. In our view, scarce staff resources may affect both the possibility of reflection on suicidal thinking and the possibility of rest from responsibility in cases of close follow-up of a patient, eventually leading to stressful situations for the caregiver as well as for the patient.

The study describes how experienced MHCPs work to take care of themselves in the encounter with suicidal patients admitted to a psychiatric unit and their family members. More research is needed, especially on how MHCPs with less work experience perceive the need for support in their encounters with suicidal behaviour. The fact that professionals manage to take care of themselves may have a positive impact on their ability to care for patients and their family members and may thus also be instrumental in preventing suicide.

## References

[cit0001] Alhamidi, S. A., & Alyousef, S. M. (2022). Perceptions of mental health nurses toward caring for suicidal hospital inpatients in Saudi Arabia. *Death Studies*, 46(5), 1166–10. 10.1080/07481187.2020.1801894 32762402

[cit0002] Bergbom, I., Nåden, D., & Nyström, L. (2022). Katie Eriksson’s caring theories. Part 1. The caritative caring theory, the multidimensional health theory and the theory of human suffering. *Scandinavian Journal of Caring Sciences*, 36(3), 782–790. 10.1111/scs.13036 34609017

[cit0003] Cutcliffe, J. R., & Barker, P. (2002). Considering the care of the suicidal client and the case for ‘engagement and inspiring hope’ or ‘observations’. *Journal of Psychiatric and Mental Health Nursing*, 9(5), 611–621. 10.1046/j.1365-2850.2002.00515.x 12358715

[cit0004] Cutcliffe, J. R., Hummelvoll, J. K., Granerud, A., & Eriksson, B. G. (2015). Mental health nurses responding to suffering in the 21st century occidental world: Accompanying people in their search for meaning. *Archives of Psychiatric Nursing*, 29(1), 19–25. 10.1016/j.apnu.2014.09.008 25634870

[cit0005] Cutcliffe, J. R., & Stevenson, C. (2008). Feeling our way in the dark: The psychiatric nursing care of suicidal people—A literature review. *International Journal of Nursing Studies*, 45(6), 942–953. 10.1016/j.ijnurstu.2007.02.002 17400229

[cit0006] Dransart, D. A. C., Treven, M., Grad, O. T., & Andriessen, K. (2017). Impact of client suicide on health and mental health professionals. In K. Andriessen, K. Krysinska, & O. T. Grad (Eds.), *Postvention in action. The international handbook of suicide handbook support* (pp. 245–254). Hogrefe Publishing.

[cit0007] Eriksson, K. (1984). *Hälsans idé. [*the idea of Health*]*. Almqvist & Wiksell.

[cit0008] Eriksson, K. (2006). *The suffering human being*. Nordic Studies Press.

[cit0009] Eriksson, K. (2018). Vårdvetenskap (caring Science*). Vetenskapen om vårdandet – om det tidlösa i tiden. Samlingsverk (the science of caritative caring – about the timeless in time*. collective work). Liber AB.

[cit0010] Gadamer, H.-G. (2004). *Truth and method* (2nd ed.). Continuum.

[cit0011] Gulfi, A., Dransart, D. A. C., Heeb, J.-L., & Gutjahr, E. (2010). The impact of patient suicide on the professional reactions and practices of mental health caregivers and social workers. *Crisis: The Journal of Crisis Intervention and Suicide Prevention*, 31(4), 202–210. 10.1027/0027-5910/a000027 20801750

[cit0012] Gustin, L. W. (2012). Medlidande (compassion. In L. W. Gustin & I. Bergbom (Eds.), Vårdvetenskapliga begrepp i teori och praktik (caring science concepts in theory and practice) (pp. 307–318). Studentlitteratur AB.

[cit0013] Hagen, J., Hjelmeland, H., & Knizek, B. L. (2017a). Relational principles in the care of suicidal inpatients: Experiences of therapists and mental health nurses. *Issues in Mental Health Nursing*, 38(2), 99–106. 10.1080/01612840.2016.1246631 27901635

[cit0014] Hagen, J., Knizek, B. L., & Hjelmeland, H. (2017b). Mental health nurses’ experiences of caring for suicidal patients in psychiatric wards: An emotional endeavor. *Archives of Psychiatric Nursing*, 31(1), 31–37. 10.1016/j.apnu.2016.07.018 28104055

[cit0015] Hendin, H., Lipschitz, A., Maltsberger, J. T., Haas, A., & Wynecoop, S. (2000). Therapists' reactions to patients' suicides. *The American journal of psychiatry*, 157(22), 2022–2027. 10.1176/appi.ajp.157.12.2022 11097970

[cit0016] Koskinen, C. A. L., & Lindström, U. Å. (2015). An envisioning about the caring in listening. *Scandinavian Journal of Caring Sciences*, 29(3), 548–554. 10.1111/scs.12149 24861609

[cit0017] Kvale, S., & Brinkmann, S. (2009). Det kvalitative forskningsintervju (the qualitative research interview) (2nd ed.). Gyldendal Akademisk.

[cit0018] Lindström, U. Å. (1994). Psykiatrisk vårdlära (textbook in psychiatric care). Liber utbildning.

[cit0019] McLaughin, C., McGowan, I., O`neill, S., & Kernohan, G. (2014). The burden of living with and caring for a suicidal family member. *Journal of Mental Health*, 23(5), 236–40. 10.3109/09638237.2014.928402 24988002

[cit0020] Mellesdal, L., Mehlum, L., Wentzel-Larsen, T., Kroken, R., & Jørgensen, H. A. (2010). Suicide risk and acute psychiatric readmissions: A prospective cohort study. *Psychiatric Services*, 61(1), 25–31. 10.1176/ps.2010.61.1.25 20044414

[cit0021] Morrissey, J., & Higgins, A. (2019). “Attenuating anxieties”: A grounded theory study of mental health nurses’ responses to clients with suicidal behaviour. *Journal of Clinical Nursing*, 28(5–6), 947–958. 10.1111/jocn.14717 30431681

[cit0022] Nåden, D. (2010). Hermeneutics and observation – a discussion. *Nursing Inquiry*, 17(1), 75–81. 10.1111/j.1440-1800.2009.00472.x 20137033

[cit0023] Scupham, S., & Goss, S. P. (2020). Working with suicidal clients: Psychotherapists and allied professionals speak perspectives on suicidology. Further reflections on suicide and psychache about their experiences. *Counselling and Psychotherapy Research*, 20(3), 525–534. 10.1002/capr.12288

[cit0024] Shneidman, E. S. (1998). Perspectives on suicidology. Further reflections on suicide and psychache. *Suicide and Life-Threatening Behavior*, 28(3), 245–250. Fall https://pubmed.ncbi.nlm.nih.gov/9807770/9807770

[cit0025] Talseth, A.-G., & Gilje, F. L. (2011). Nurses’ responses to suicide and suicidal patients: A critical interpretive synthesis. *Journal of Clinical Nursing*, 20(11–12), 1651–1667. 1651 10.1111/j.1365-2702.2010.03490.x 21366737

[cit0026] Türkles, S., Yilmas, M., & Soylu, P. (2018). Feelings, thoughts and experiences of nurses working in a mental health clinic about individuals with suicidal behaviors and suicide attempts. *Collegian (Royal College of Nursing, Australia)*, 25(4), 441–446. 10.1016/j.colegn.2017.11.002

[cit0027] Vatne, M. (2006). Psykiatriske sykepleieres forståelse av eget ansvar i arbeid med selvmordsnære pasienter (psychiatric nurses’ understanding of their responsibility working with suicidal patients). *Värd I Norden*, 26(1), 30–35. 10.1177/010740830602600107

[cit0028] Vatne, M., Lohne, V., & Nåden, D. (2021). “Embracing is the most important thing we can do” – caring for the family members of patients at risk of suicide. *International Journal of Qualitative Studies on Health and Well-Being*, 16(1). 10.1080/17482631.2021.1996682 PMC8635556 34806566

[cit0029] Vråle, G. B. (2014). Bekymringer som blir bekreftet – om helsepersonells opplevelser når en pasient tar sitt eget liv (concerns that are confirmed - about health personnel’s experiences when a patient takes their own life). *Tidsskrift for Psykisk Helsearbeid*, 11(1), 3–12. 10.18261/ISSN1504-3010-2014-01-02

[cit0030] WHO, World Health Organization. (2023, August 28). Suicide. Suicide (who.int). Suicide (who.int).

[cit0031] Williams, J. M. G. (2014). *Cry of pain. Understanding suicide and the suicidal mind*. Piatkus.

